# O02 Coccidioidomycosis infection: a clinical conundrum

**DOI:** 10.1093/jacamr/dlaf230.002

**Published:** 2025-12-04

**Authors:** Amy Carroll, Daniel Stevenson, Jonathan Lambourne

**Affiliations:** Barts Health NHS Trust, London, UK; Barts Health NHS Trust, London, UK; Barts Health NHS Trust, London, UK

## Abstract

**Background:**

Coccidioidomycosis is a fungal infection caused by *Coccidioides immitis* and *Coccidioides posadasii*. These soil-dwelling dimorphic fungi are primarily found in arid environments in Central/Southern California, several Southwestern states and parts of Central and South America.^1^ Human infection occurs through inhalation of arthroconidia, resulting in a respiratory illness in one-third of exposed cases. Haematogenous spread from the lung can result in infection at extra-pulmonary sites, most commonly skin, musculoskeletal and central nervous system.^1,2^ Most infections resolve without treatment, however antifungal therapy (usually with an azole) is indicated in some patient groups.^1–3^ We report a case of *Coccidioides* pulmonary infection following travel to an endemic area in a comorbid patient.

**Case presentation:**

A 55-year-old Caucasian female presented with a 2 week history of dry cough and 2 day history fever and myalgia 2 days after return from a 11 day holiday in North America, which involved travel to several Southwestern states including Arizona, Nevada, Texas and New Mexico. Her past medical history included granulomatosis with polyangiitis (on long-term prednisolone 5–10 mg/day), asthma (on benralizumab) and bronchiectasis. She was admitted and treated empirically for community-acquired pneumonia with piperacillin/tazobactam. Chest radiograph showed new infiltrates left mid and lower zones. She clinically improved and was discharged on Day 5. In light of the travel history, Coccidioides serology was sent. Coccidioides IgG (by enzyme-linked immunoassay) was reported as indeterminate and a repeat sample sent six weeks later was positive, diagnostic for Coccidioides infection. Extended sputum culture on Sabouraud agar at 30°C and 37°C did not yield growth of *Coccidioides* species. She remained well and an interval computed tomography scan showed resolution of the left-sided consolidation with a small residual area of nodularity.

**Discussion:**

Our case illustrates the common natural course of Coccidioides infection. Guidelines recommend that all patients should be monitored for two years after the acute infection to ensure illness remains uncomplicated and monitor serology and radiographic changes.^1^ Pulmonary nodules or cavities are common sequelae following Coccidioidal pneumonia and treatment is generally not advised, unless the patient is symptomatic.^1,2^ This patient’s rheumatological condition adds complexity to the management. Coccidioides can remain dormant for years after the initial infection and can reactivate when T-cell mediated immunity is impaired, for example due to high-dose corticosteroids or biological response modifier (BRM) therapy. There is a lack of consensus around BRM usage and Coccidioides infection, particularly patients who are asymptomatic with positive serologies in whom the need for prophylaxis or suppressive treatment is unclear.^1,3^ Benralizumab is an IL-5 inhibitor, which has no reported increase in the risk of endemic fungal infections based on available clinical data.^4^ As such, in her case the risk of reactivation would increase if immune suppression is escalated to include cell-mediated (CD4+) and tumour necrosis factor alpha pathways.^1,3^

**Conclusions:**

Our case highlights the importance of diagnosing and closely monitoring Coccidioides infection among patients on immunosuppressive therapies, who are at increased risk of disseminated infection and disease reactivation.
